# Characterization of Non-Covalent Interactions Between Whey Protein and Tannic Acid

**DOI:** 10.3390/foods15214004

**Published:** 2026-11-09

**Authors:** Han Song, Zengjia Zhou, Jin Wu, Yuehan Zeng, Yuwen Li, Gege Hu, Zhuoting Zhang, Bingyu Yang, Huimei Xia, Haoda Shu, Jinghao Yang, Wen Zhao, Justyna Zulewska, Shaoli Liu, Jian Zhang

**Affiliations:** 1 School of Food and Health, Beijing Technology and Business University, Beijing 100048, China; songh0107@163.com (H.S.); m15255825668@163.com (Z.Z.); 17681327631@163.com (J.W.); zeng55272024@163.com (Y.Z.); 18407718130@163.com (Y.L.); zzt17303410361@163.com (Z.Z.); 19835230803@163.com (B.Y.); zzzs112277@163.com (H.X.); shuhaoda123@outlook.com (H.S.); haah1212@163.com (J.Y.); 2 Key Laboratory of Geriatric Nutrition and Health, Beijing Technology and Business University, Ministry of Education, Beijing 100048, China; hugege0819@163.com; 3 School of Light Industry Science and Engineering, Beijing Technology and Business University, Beijing 100048, China; 4 Inner Mongolia Dairy Technology Research Institute Co., Ltd., Hohhot 010110, China; 13614317400@163.com; 5 School of Food Technology and Nutrition, University of Warmia and Mazury in Olsztyn, 10-719 Olsztyn, Poland; justyna.zulewska@uwm.edu.pl; 6 College of Biological and Chemical Engineering, Zhejiang University of Science and Technology, Hangzhou 310023, China; shaolil@126.com

**Keywords:** tannic acid, whey protein isolate, non-covalent, functional properties

## Abstract

Whey protein and tannic acid (TA) are important functional components that have been extensively studied, but the concentration-dependent effects of TA on the physicochemical characteristics and functional properties of WPI remain insufficiently characterized. Here, whey protein isolate (WPI, 10 mg/mL) and TA at various concentrations (0–2 mg/mL) were employed to prepare WPI-TA complexes, which were studied by different analytical techniques. In comparison with WPI, WPI–TA association resulted in concentration-dependent changes in the overall UV absorption characteristics of the system. FTIR analysis further indicated changes in the spectral characteristics associated with WPI–TA interactions after TA addition, although the contribution of TA-related absorption could not be completely excluded. The results suggested that hydrogen bonding may contribute to WPI–TA association, although the involvement of other non-covalent interactions cannot be excluded. The reduced apparent free sulfhydryl content in WPI–TA complexes suggested changes in the sulfhydryl environment or the accessibility of sulfhydryl groups in WPI. Furthermore, WPI–TA interactions led to increased particle size, significantly (*p* < 0.05) improved emulsifying and foaming properties, and alterations in the thermal transition behavior of the protein. Therefore, this study offers an effective approach to the modification of whey protein by polyphenols to obtain functional ingredients for potential application in functional foods.

## 1. Introduction

Whey, a by-product of cheese processing, is rich in several important nutrients, including whey proteins, which are composed mainly of globular proteins including β-lactoglobulin, α-lactalbumin, bovine serum albumin, immunoglobulins and lactoferrin [1]. Whey protein isolate (WPI) is the isolated form of whey protein, which is manufactured by removing a significant amount of non-protein components from whey through membrane separation, ion-exchange chromatography, or microfiltration methods, followed by concentration and spray-drying [2,3]. WPI has been characterized with superior nutritional value, with enhanced digestibility and absorption, and it has been widely utilized in various forms of food-processing owing to its advantageous functional attributes, including solubility, foaming, emulsification, and gelation [4,5,6]. However, WPI is susceptible to oxidation during processing, negatively affecting its functional properties and biological activities [7]. A multitude of studies have concentrated on enhancing the functional attributes of WPI through physical and chemical alterations in its structure [8,9,10], but there are shortcomings, such as elevated costs and the introduction of chemical reagents that can easily cause food safety problems [11]. Consequently, searching for a low-cost and non-toxic reagent, together with an efficient strategy to strengthen the functional features of WPI, has become a significant research challenge.

Polyphenols are a class of bioactive substances with at least one aromatic ring and multiple hydroxyl or substituent groups, which are present in fruits, vegetables, dried fruits, beverages, legumes, cereals, herbs and spices [12,13]. Polyphenols are primarily categorized as phenolic acids, flavonoids, stilbenes, lignans and others, which have been reported as having various bioactivities, including anti-inflammation, antitumor, antioxidation, and antivirus [14]. Interactions between polyphenols and proteins through covalent or non-covalent binding were found to modify the structural and functional attributes of proteins [15]. The protein–polyphenol covalent conjugates formed by alkaline, radical grafting and enzyme-catalyzed reactions were shown to have higher stability and better functional properties [16,17]. However, the protein–polyphenol conjugates exhibited lower digestibility and bioavailability due to the permanent covalent binding, and enzymatic preparation of these conjugates was complicated and expensive [18,19]. Unlike covalent binding, non-covalent interactions between proteins and polyphenols are reversible and may involve hydrogen bonding, hydrophobic interactions, electrostatic interactions, and van der Waals forces, depending on factors such as pH, ionic strength, protein charge, and polyphenol structure [20,21,22]. It was reported that the non-covalent combination of WPI with three polyphenols improved solubility, emulsification and foaming properties of WPI [23]. Formation of resveratrol and WPI complexes by hydrogen bonding and hydrophobic interactions increased the emulsifying and foaming abilities of WPI [24].

Tannic acid (TA) is a water-soluble polyphenol with excellent antioxidant, anti-inflammatory, antibacterial, and antitumor properties, which is widely found in the bark of oak, chestnut, hemlock, and mangrove trees, as well as in the leaves of specific lacquer trees and plant galls [25,26]. It is widely used in emulsions, hydrogels, and films [27,28]. TA belongs to the class of hydrolysable gallotannins, which are composed of a sugar core esterified with gallic acid-derived units [29]. Commercial TA preparations may exhibit structural diversity due to variations in galloyl glucose structures and galloylation degrees, and their abundant phenolic hydroxyl groups can participate in protein interactions through various noncovalent forces [30,31]. It was reported that formation of a non-covalent complex between TA and soybean protein isolate through hydrogen bonding altered the structure of the protein, with enhanced emulsification properties [19]. TA also formed complexes with milk proteins through hydrophobic interactions, resulting in an enhancement in the foaming capacity of lactoferrin and thermal stability of bovine serum albumin [32,33]. Previous studies have investigated WPI–TA interactions under different experimental conditions, looking at their aggregation behavior, binding characteristics, and interfacial or functional properties [22,34,35]. However, the combined concentration-dependent effects of non-covalently prepared WPI–TA complexes on thermal stability, emulsifying properties, and foaming properties remain insufficiently characterized.

In the present work, non-covalent complexes of WPI and TA were formed by binding varying levels of TA to WPI, and the structural changes in WPI were analyzed by ultraviolet absorption spectroscopy and Fourier transform infrared spectroscopy. The non-covalent WPI-TA complexes were further characterized by particle size, total free sulfhydryl groups, TA binding and binding quantity. The effects of TA addition on the thermal stability, foaming characteristics, and emulsification properties of WPI were also assessed. This study aimed to systematically evaluate the concentration-dependent structural and functional responses of WPI to commercial TA, with particular emphasis on thermal stability, emulsifying properties, and foaming performance.

## 2. Materials and Methods

### 2.1. Materials

WPI in powder form with a purity of 92.76% was purchased from Hilmar Ingredients (Hilmar, CA, USA), and commercial TA powder (CAS No:1401-55-4, supplier-reported purity ≥ 95%, analytical-grade) from Aladdin Co., Ltd. (Shanghai, China). Commercial TA is a heterogeneous mixture of galloyl ester derivatives rather than a single compound. The detailed galloyl ester composition and average degree of galloylation of the specific TA preparation used in this study were not further characterized. Sunflower oil (Arawana brand, Yihai Kerry Arawana Holdings Co., Ltd., Shanghai, China) was purchased from a local supermarket in Beijing, China. All other chemical agents used were of analytical grade.

### 2.2. Preparation of WPI-TA Complex Samples

The non-covalent WPI-TA complex samples were prepared following the procedure of Wang et al. [19] with a few changes. Briefly, WPI solution (10 mg/mL) was prepared by solubilization of WPI powder in deionized water with constant stirring (500 rpm) for 2 h at room temperature, and kept at 4 °C for further use. The WPI-TA complexes at TA concentrations of 0.25, 1.00, and 2.00 mg/mL, respectively, were then prepared at room temperature by the addition of certain amount of TA powder to the WPI solution, which was adjusted to pH 7.0. These TA concentrations were selected based on previous studies regarding protein–TA non-covalent complexes, in which TA levels in the mg/mL range were commonly used to investigate protein–polyphenol interactions and their effects on functional properties [19,36]. The mixture was stirred for 2 h (500 rpm) in an anoxic environment to obtain the WPI-TA non-covalent complex samples, named N-WPITA1, N-WPITA2, and N-WPITA3, in ascending order of TA concentration. The resultant complexes were further dialyzed at 4 °C for 24 h using dialysis bags (7 kDa molecular weight cutoffs) with three changes in distilled water as the external dialysis medium. The external dialysis volumes were 250, 700, and 700 mL for the initial TA concentrations of 0.25, 1, and 2 mg/mL, respectively. The samples were then lyophilized and stored at 4 °C for subsequent analyses. Natural WPI (10 mg/mL) without the addition of TA (N-WPI) was also prepared following the same procedure described above and used as a control sample.

### 2.3. Ultraviolet (UV) Absorption Spectroscopy

The UV absorption spectra of the samples (N-WPI, N-WPITA1, N-WPITA2, and N-WPITA3) were measured by using UV-3600 ultraviolet–visible–near-infrared spectrophotometer (Shimadzu, Kyoto, Japan) in accordance with the method of Wang et al. [19]. The N-WPI and WPI-TA non-covalent complexes in powder form were dissolved in phosphate-buffered saline (PBS, pH 7.0) to attain a level of 0.2 mg/mL for the analyses within the wavelength range of 230–500 nm at room temperature. PBS was used as a blank for baseline calibration. TA-alone solutions at concentrations of 0.25, 1.00, and 2.00 mg/mL were also prepared and measured separately as reference controls. These concentrations corresponded to the initial TA concentrations used in the preparation of the WPI–TA complexes, allowing for comparison of the UV absorption characteristics of TA alone and the WPI–TA systems.

### 2.4. Fourier Transform Infrared (FTIR) Spectroscopy

Freeze-dried N-WPI WPI–TA complex, and TA samples were mixed with potassium bromide (KBr) at a ratio of approximately 1:100 (*w*/*w*), pressed into transparent pellets, and analyzed by FTIR spectroscopy (Nicolet iS10, Thermo Fisher, Madison, WI, USA) in transmission mode at room temperature [37]. The spectrometer had a signal-to-noise ratio of 50,000:1. Spectra were recorded over 400–4000 cm^−1^ at a resolution of 4 cm^−1^, with 32 scans per spectrum. Baseline correction was performed using PeakFit v4.12 software. The FTIR spectra were analyzed to evaluate spectral changes associated with WPI–TA interactions. The resulting spectra were plotted using Origin 2021 software.

### 2.5. Measurement of TA Binding Ratio and Binding Amount

The Folin–Ciocalteu assay was used to quantify the amount of TA released into the dialysis medium during dialysis. The external dialysis solutions from three dialysis cycles were collected and analyzed. The amount of TA retained in the WPI–TA system after dialysis was estimated using a mass balance approach between the initial TA added and the diffusible TA quantified in the external dialysis solutions [23]. Since WPI molecules were retained inside the dialysis membrane (7 kDa MWCO), the Folin response detected in the dialysis medium mainly represented diffusible TA. Briefly, 0.5 mL of sample solution was mixed with 0.5 mL of Folin–Ciocalteu reagent (0.25 mol/L) in the same volumes and reacted for 3 min in the dark. Then, 1 mL of Na_2_CO_3_ solution (15%, *w*/*v*) was added, and the mixture was further reacted for 30 min in the dark. The absorbance at 760 nm was determined by an Infinite 200 Pro microplate reader (Tecan, Shanghai, China) using a reagent blank containing all reagents except the sample for background correction, and different concentrations of TA solution were used to establish the standard curve. The TA binding ratio and amount of TA bound per milligram of WPI were calculated using the equations below:

$$\mathrm{TA}\;\mathrm{binding}\;\mathrm{ratio}\;(\%)\;=\frac{\mathrm{total TA}-\mathrm{TA dialyzed}}{\mathrm{total TA}}\times 100\%$$

The mg of TA binding per mg of WPI=total TA mg×TA binding ratio (%)mg of WPI ×100

A TA control at the highest TA concentration used in this study (2.0 mg/mL) was used to evaluate the efficiency of dialysis for removing freely diffusible TA. The control was subjected to the same dialysis procedure described above, and three independently prepared TA samples were analyzed. The TA concentrations before and after dialysis were determined using the Folin–Ciocalteu assay described in Section 2.5. The TA removal ratio was calculated from the residual TA remaining inside the dialysis bag using the equation below:

$$\mathrm{TA}\;\mathrm{removal}\;\mathrm{ratio}\;(\%)\;=\frac{C_{0}-C_{f}}{C_{0}}\times 100$$

where C_0_ is the TA concentration before dialysis and C_f_ is the TA concentration remaining inside the dialysis bag after dialysis.

### 2.6. Determination of Particle Size

The N-WPI and WPI-TA complex samples were dissolved in PBS (pH 7.0) to achieve a sample concentration of 1 mg/mL. The Z-average diameter (DZ, intensity-weighted mean diameter) and polydispersity index (PDI) were determined using a Zetasizer Nano ZS90 (Malvern Instruments Ltd., Malvern, Worcestershire, UK) at 25 °C according to the method of Han et al. [38]. The refractive indexes of the dispersed medium and protein were 1.33 and 1.46, respectively. In addition, TA-alone solutions at 0.25, 1.00, and 2.00 mg/mL were analyzed under the same conditions to evaluate the contribution of TA self-association to the particle-size behavior of the WPI–TA systems.

### 2.7. Determination of Differential Scanning Calorimetry (DSC)

The DSC curves of N-WPI, TA, and WPI-TA complexes were determined by differential scanning calorimeter (DSC1, Mettler Toledo, Greifensee, Switzerland), basically following the procedure of Boachie et al. [39]. Each N-WPI, TA, and WPI-TA complex sample in powder form (5 mg) was loaded with a sealed aluminum dish. An empty aluminum dish was regarded as a reference, and nitrogen was employed as carrier gas at a nitrogen flow rate of 50 mL/min. The samples were equilibrated for 10 min at 25 °C, followed by heating to 250 °C at a heating rate of 10 °C/min. Three independently prepared samples were analyzed for each group (*n* = 3). The onset temperature (T_onset_) and peak temperature (T_max_) of each thermal transition were determined from the DSC thermograms.

### 2.8. Measurement of Free Sulfhydryl (SH) Group

The contents of the SH group in the N-WPI and WPI-TA complexes were measured following the description by Meng et al. [23]. In addition, TA-alone solutions at 0.25, 1.00, and 2.00 mg/mL were subjected to the same assay conditions to evaluate the potential contribution of TA itself to the absorbance at 412 nm. The absorbance contribution of TA was not subtracted from that of the WPI–TA samples. A reagent blank containing all assay reagents except the sample was used for background correction. Three independently prepared TA-alone samples were analyzed at each concentration. The Tris-Gly solution was prepared by adding 10.4 g of Tris(hydroxymethyl) aminomethane, 6.9 g of glycine, and 1.2 g of ethylene diamine tetraacetic acid in 1 L of deionized water adjusted to pH 8.0. The Ellman’s reagent was prepared by dissolving 0.02 g of 5,5-dithiobis nitro-benzoic acid (DTNB) in 5 mL of Tris-Gly solution, corresponding to a DTNB concentration of 4 mg/mL. The freeze-dried N-WPI and WPI–TA samples were redissolved in PBS (pH 7.0) to obtain a sample concentration of 10 mg/mL. Each N-WPI and WPI-TA complex sample (0.5 mL) was added to Tris-Gly solution containing 2.5 mL of 8 mol/L urea and 20 μL of Ellman’s reagent. After reaction for 60 min at room temperature, the absorbance at 412 nm was determined by an ultraviolet spectrophotometer (Cary 60, Agilent Technologies, Penang, Malaysia), and the free SH group content was calculated as

$$\mathrm{SH}\;(\mu \mathrm{mol}/g)=\frac{73.53\times A_{412}\times D}{C}$$

where 73.53 is the conversion coefficient derived from 10^6^/(1.36 × 10^4^); 1.36 × 10^4^ M^−1^ cm^−1^ is the molar absorption coefficient and 10^6^ is used for conversions from mole to micromole and from milligram to gram; A_412_ is the absorbance at 412 nm; D is the dilution factor; and C is the sample concentration (mg/mL).

### 2.9. Measurement of Emulsifying Ability and Emulsion Stability

The emulsifying activity index (EAI) and emulsifying stability index (ESI) of the N-WPI and WPI-TA complexes were assessed following the description of Li et al. [40] with slight variations. Briefly, the N-WPI and WPI-TA complexes were dissolved in PBS (pH 7.0) to achieve a protein concentration of 2 mg/mL. Sunflower oil (5 mL) was mixed with N-WPI or WPI-TA aqueous dispersions (15 mL) to form emulsions using a high-speed homogenizer (Ultra Turrax T25, IKA Labortechnik, Staufen, Germany) at 10,000 rpm for 1 min. The emulsions were then kept standing. Samples (50 μL) were collected from the bottom of the tube at 0 and 10 min of standing and diluted 100 times with 0.1% (*w*/*v*) sodium dodecyl sulfate. The absorbance of the diluted emulsion was determined at 500 nm using an ultraviolet spectrophotometer (Cary 60, Agilent Technologies, Malaysia). EAI and ESI were calculated as

$$\mathrm{EAI}\;(m^{2}/g)=\frac{2\times 2.303\times A_{0}\times \mathrm{DF}}{C\times L\times \Phi \times 10,000}$$

$$\mathrm{ESI}\;(\min)=\frac{A_{0}}{A_{0}-A_{10}}\times 10$$

where DF is the dilution factor; C is the protein concentration (g/mL); L is the optical path (1 cm); and Φ is the oil volume fraction (0.25). A_0_ and A_10_ refer to the absorbance of the emulsion at 0 and 10 min, respectively.

### 2.10. Measurement of Foam Capacity and Foam Stability

The foam capacity (FC) and foam stability (FS) of N-WPI and WPI-TA aqueous dispersions were determined using a high-speed homogenizer (Ultra Turrax T25, IKA Labortechnik, Germany) according to the method described by Deng et al. [41]. In brief, the N-WPI and WPI–TA samples were dispersed in deionized water at a concentration of 10 mg/mL (based on the total mass of freeze-dried sample powder). Then, 20 mL of each dispersion was homogenized at 10,000 rpm for 1 min, and the foam volumes were recorded at 0 and 30 min, respectively. FC and FS were calculated as

$$\mathrm{FC}\;(\%)=\frac{V_{0}}{V}\times 100$$

$$\mathrm{FS}\;(\%)=\frac{V_{30}}{V_{0}}\times 100$$

where V_0_, V_30_, and V represent the foam volumes at 0 and 30 min, as well as the initial aqueous dispersion volume, respectively.

### 2.11. Statistical Analysis

Measurements of TA binding ratio and binding amount, particle size, FTIR-derived spectral shifts, DSC thermal transition parameters (T_onset_ and T_max_), free sulfhydryl content, emulsifying properties, and foaming properties were performed using three independently prepared WPI–TA samples (*n* = 3). The results are expressed as mean ± standard deviation, and one-way analysis of variance (ANOVA) followed by Duncan’s test at *p* < 0.05 was applied where appropriate. Measurements performed only once were presented descriptively and were not included in statistical analysis.

## 3. Results

### 3.1. UV Spectroscopy Analysis

**Figure 1:**
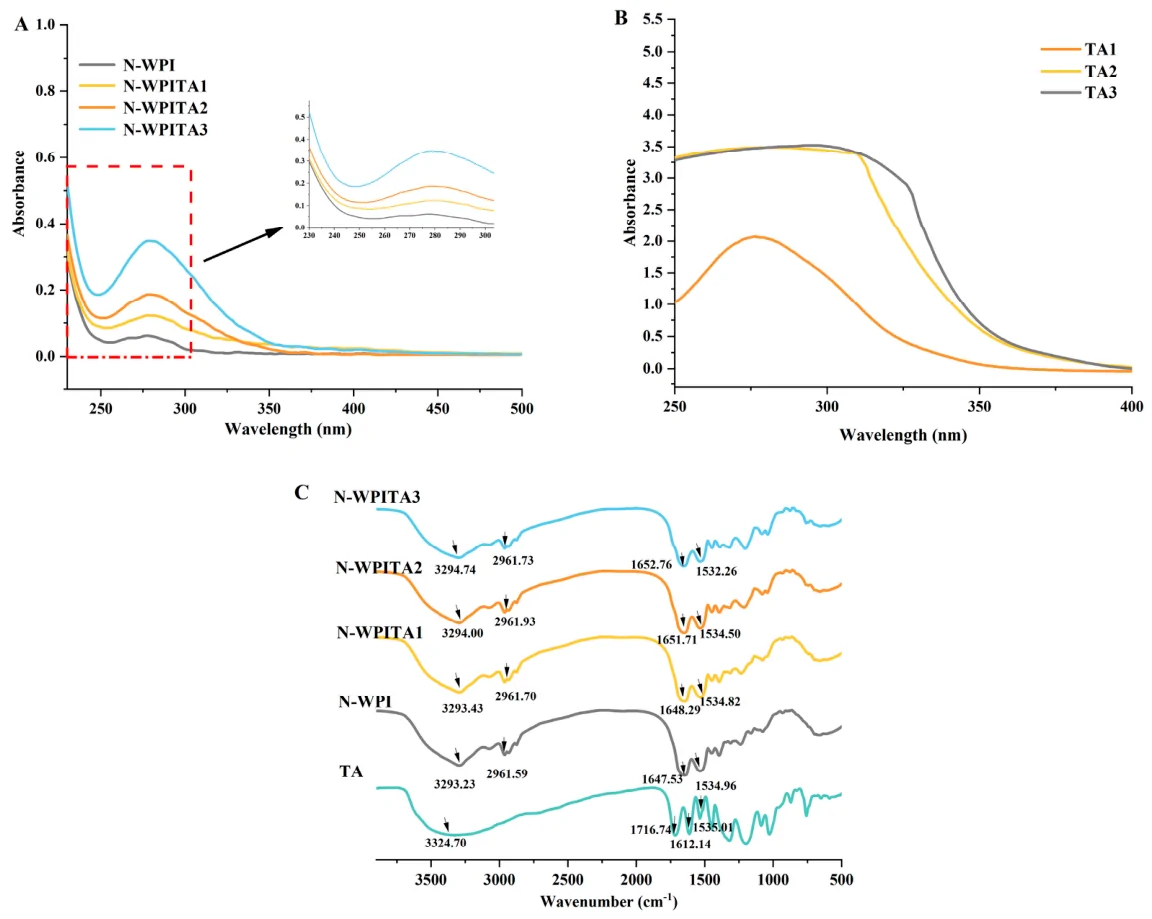
UV spectra of N-WPI and WPI–TA samples (**A**), UV spectra of TA alone at different concentrations (**B**), and FTIR spectra of N-WPI, WPI–TA complexes, and TA (**C**). N-WPI: WPI at 10 mg/mL; N-WPITA1: WPI at 10 mg/mL with TA at 0.25 mg/mL; N-WPITA2: WPI at 10 mg/mL with TA at 1 mg/mL; N-WPITA3: WPI at 10 mg/mL with TA at 2 mg/mL. TA concentrations in panel (**B**) were 0.25, 1.00, and 2.00 mg/mL. The red dashed box in (**A**) indicates the region selected for magnification, and the black arrow points to the corresponding magnified inset.

Ultraviolet–visible absorption spectroscopy is a commonly used technique to study the interaction between proteins and polyphenols, as well as changes in the overall spectral characteristics of protein–polyphenol systems [24]. Variations in absorbance intensity may reflect changes in the overall UV absorption characteristics of protein–polyphenol systems, whereas shifts in peak position may provide information related to changes in the microenvironment of aromatic residues [42]. As shown in Figure 1A, there were strong characteristic absorption peaks with different intensities near 280 nm in the UV absorption spectra of the N-WPI and WPI-TA complexes, which are mainly associated with the absorption of aromatic amino acid residues in proteins, such as tyrosine and tryptophan [43]. Differences in absorbance intensity were observed among WPI–TA complexes with increasing TA concentrations. However, TA-alone controls exhibited strong UV absorption in the same wavelength region (Figure 1B), indicating that TA itself exhibits considerable UV absorption and substantially contributes to the overall spectral response of the WPI–TA system. Therefore, the UV absorbance changes cannot be solely attributed to alterations in the exposure environment of tryptophan and tyrosine residues. Furthermore, slight shifts in the characteristic absorption positions of the WPI–TA complexes were observed with increasing TA concentrations, which may indicate changes in the local environment of aromatic residues after TA incorporation; however, the contribution of TA-associated absorption cannot be completely excluded [44,45]. Similar increases in the characteristic UV absorption intensity and red shifts in the peak positions were also reported earlier for the non-covalent complexes formed by soybean protein isolate and tannic acid [19,46]. Thus, the UV results mainly reflected the combined optical response of WPI–TA systems, while possible conformational alterations were further evaluated using FTIR analysis.

### 3.2. FTIR Analysis

FTIR is a commonly used technique for characterizing protein-associated spectral changes and providing information about possible interactions between proteins and polyphenols [43]. In the infrared spectrum, the amide I band (1600–1700 cm^−1^), which is primarily associated with C=O stretching of peptide bonds, and the amide II band (1500–1600 cm^−1^), which indicates the coupling of C-N stretching vibrations with N-H bending modes, are commonly used for evaluating protein conformational characteristics [47]. The amide A band (3200–3300 cm^−1^) indicates the stretching vibration of the O-H and N-H bonds, while the absorption peak at 2800–3000 cm^−1^ is ascribed to the C-H stretching vibration [48,49]. Figure 1C displays the FTIR spectra of N-WPI and WPI-TA complexes. The amide I and amide II bands changed from 1647.53 and 1534.96 cm^−1^ in N-WPI to 1652.76 and 1532.26 cm^−1^ in N-WPITA3, respectively. These spectral changes may reflect changes in the overall spectral environment of the WPI–TA system after TA addition, although the influence of TA absorption should also be considered. Similar shifts in the amide I and amide II bands have also been reported in non-covalent whey protein concentrate–quercetin complexes and were associated with changes in protein-associated spectral characteristics [50]. N-WPI exhibited a band at 3293.23 cm^−1^, whereas only minor variations were observed for the WPI–TA complexes in this region. TA alone exhibited a broad band at 3324.70 cm^−1^. Considering the small magnitude of these variations, changes in this region were interpreted cautiously. Hydrogen bonding may contribute to WPI–TA association [37], but the FTIR results alone are insufficient to establish it as the predominant interaction. Shifts in the characteristic peak in the amide A band were also observed for the non-covalent complexes formed by soy protein isolate and catechin when compared with the soy protein isolate [51]. In addition, N-WPI presented a distinct absorption peak at 2961.59 cm^−1^, whereas the peak positions of the WPI-TA complexes were barely changed, suggesting that no obvious changes in aliphatic C–H stretching vibrations were detected under the present experimental conditions. Therefore, the involvement of hydrophobic interactions cannot be determined solely from the FTIR results. However, electrostatic interactions and van der Waals forces cannot be excluded, as the relative contribution of different non-covalent interactions may vary with pH, ionic strength, protein charge, and polyphenol structure. Considering the overlapping absorption of TA in the amide I region, the observed spectral changes were interpreted as overall spectral variations associated with WPI–TA interactions rather than definitive structural alterations in WPI. The peak positions discussed above were obtained from the representative FTIR spectra shown in Figure 1C, whereas the values in Table 1 represent the mean ± standard deviation of three measurements.

### 3.3. Thermal Stability Analysis

**Figure 2:**
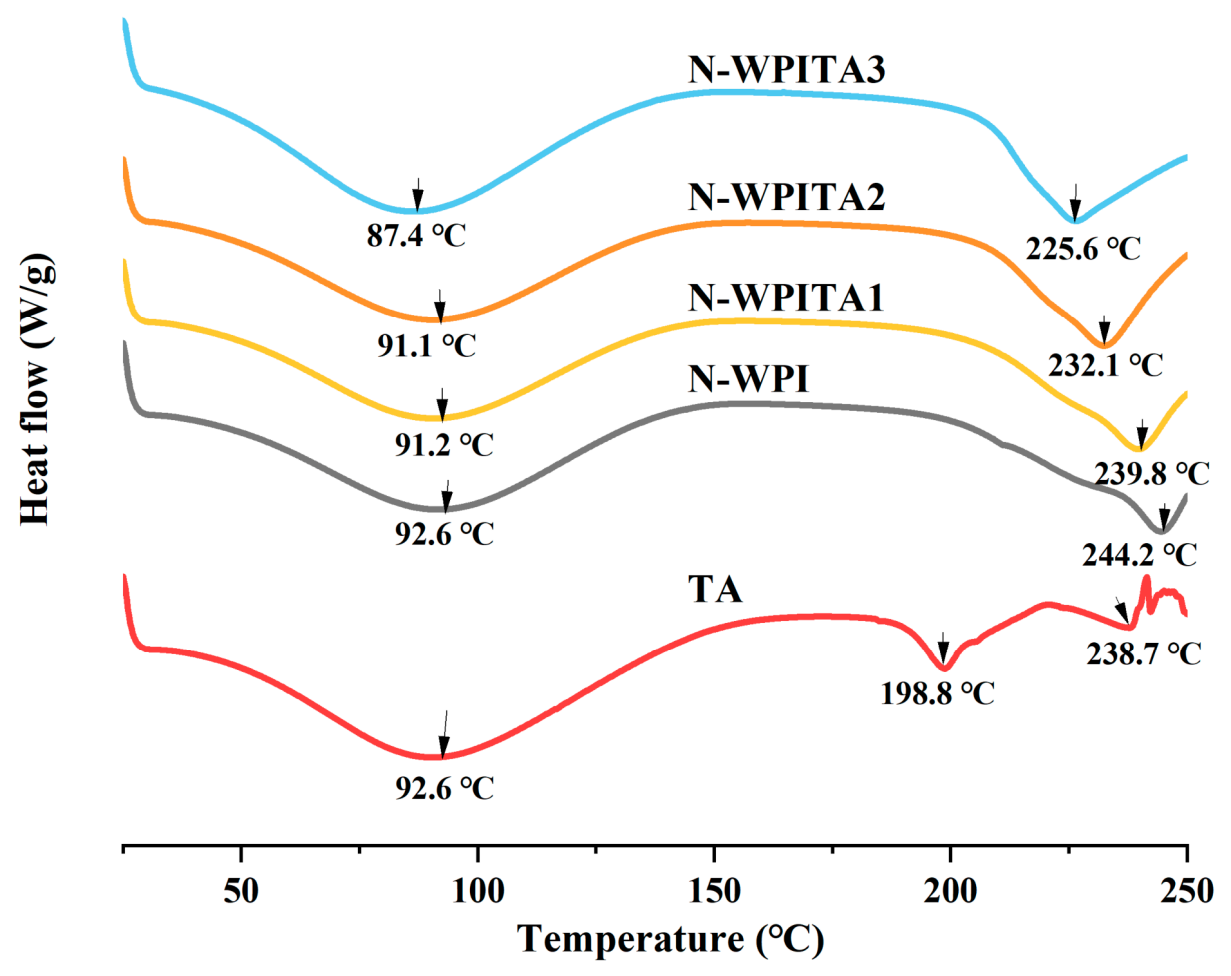
Thermal properties of N-WPI and WPI-TA complexes. N-WPI: WPI at 10 mg/mL; TA: tannic acid; N-WPITA1: WPI at 10 mg/mL, TA at 0.25 mg/mL; N-WPITA2: WPI at 10 mg/mL, TA at 1 mg/mL; N-WPITA3: WPI at 10 mg/mL, TA at 2 mg/mL.

DSC quantifies the molar heat capacity of a sample in relation to temperature, and the thermal denaturation of proteins causes conformational alterations and breakage of chemical bonds that are endothermic, while the disruption and aggregation of hydrophobic interactions are exothermic [52,53]. Figure 2 displays the DSC curves of TA, N-WPI and WPI-TA complexes. The corresponding onset temperatures (T_onset_) and peak temperatures (T_max_) are summarized in Table 2. All samples showed similar DSC curves except TA, which had two endothermic peaks at 198.8 °C and 238.7 °C, probably due to the breakdown of TA, and one endothermic peak at 92.6 °C, attributed to its water evaporation [54]. In comparison with N-WPI with an endothermic peak at 244.2 °C, the WPI-TA complexes had decreased temperatures for the peaks at 239.8 °C (N-WPITA1), 232.1 °C (N-WPITA2), and 225.6 °C (N-WPITA3), which might be attributed to the increased loss of binding water with greater proportions of TA in the WPI-TA complexes [55]. Statistical analysis showed that T_onset_, 1 did not differ significantly among N-WPI and WPI-TA samples (*p* = 0.756), whereas T_max_, 1 was significantly decreased in N-WPITA3 (*p* < 0.05). More pronounced changes were observed for the second thermal transition, with both T_onset_, 2 and T_max_, 2 decreasing significantly after TA addition (*p* < 0.05). Furthermore, the changes in the endothermic peaks in the range of 80–100 °C and 220–250 °C suggested alterations in intermolecular interactions and protein structural organization [53]. The reduced temperatures of the endothermic peaks from 92.6 °C (N-WPI) to 87.4 °C (N-WPITA3), and from 244.2 °C (N-WPI) to 225.6 °C (N-WPITA3), indicated that TA incorporation altered the thermal transition behavior and molecular organization of WPI. Therefore, non-covalent interactions between WPI and TA particularly affected the high-temperature thermal transition of WPI.

### 3.4. Particle Size Analysis

**Figure 3:**
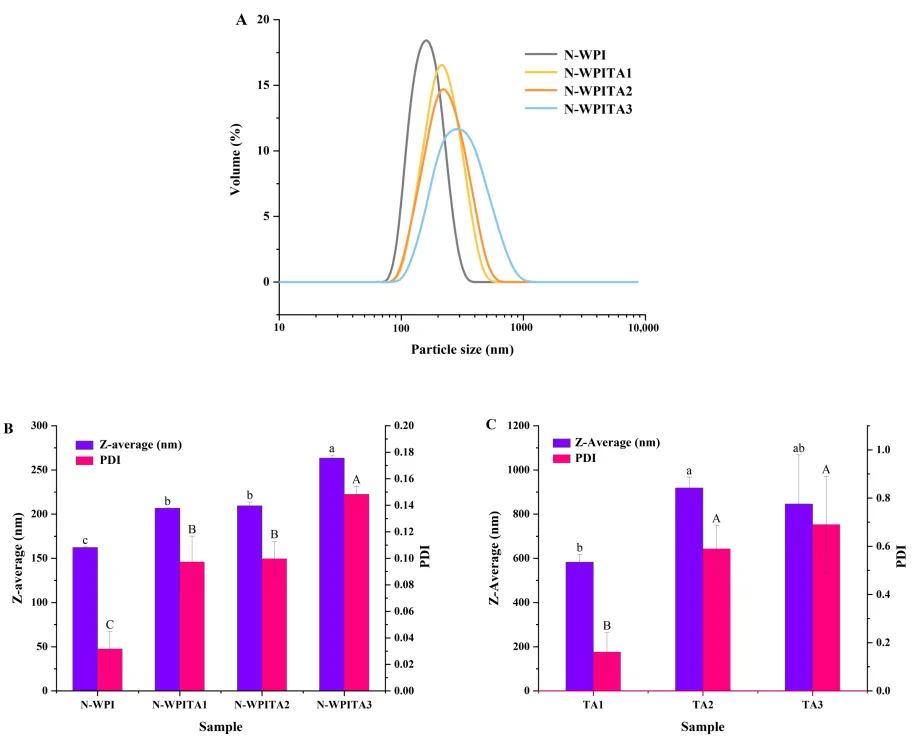
Particle volume distribution (**A**), Z-average diameter (D_Z_, intensity-weighted mean diameter), and polydispersity index (**B**) of N-WPI and WPI-TA complexes. N-WPI: WPI at 10 mg/mL; N-WPITA1: WPI at 10 mg/mL, TA at 0.25 mg/mL; N-WPITA2: WPI at 10 mg/mL, TA at 1 mg/mL; N-WPITA3: WPI at 10 mg/mL, TA at 2 mg/mL. Z-average diameter (D_Z_, intensity-weighted mean diameter), and polydispersity index (**C**) of TA samples at 0.25 mg/mL (TA1), 1.00 mg/mL (TA2), and 2.00 mg/mL (TA3). Different lowercase letters (a–c) and uppercase letters (A–C) indicate significant differences in Z-average and PDI among samples, respectively (*p* < 0.05).

Particle size and polydispersity index (PDI) provide information on the colloidal size and size-distribution heterogeneity of dispersed systems [56,57]. Changes in the volume distribution curves, Z-average diameter, and PDI of N-WPI and WPI-TA complexes are shown in Figure 3. Both N-WPI and WPI-TA complexes showed single peaks in distribution within the range of 100–1000 nm, and the peak positions shifted toward larger particle sizes with increasing proportions of TA in the WPI-TA complexes (Figure 3A). The Z-average diameters of N-WPI, N-WPITA1, N-WPITA2, and N-WPITA3 were 162.2 nm, 206.7 nm, 209.3 nm, and 263.4 nm, respectively (Figure 3B), indicating that the incorporation of commercial TA preparation affected the particle size distribution of the WPI-based system. The additional TA measurements showed substantially larger apparent Z-average diameters of 582.3, 918.0, and 845.5 nm for TA1, TA2, and TA3, respectively, with corresponding PDI values of 0.161, 0.589, and 0.690. These results suggest that TA itself underwent considerable self-association under the measurement conditions and may also contribute to the observed particle size behavior of the WPI–TA systems [58]. Similarly increased particle sizes of the complexes formed by hemp seed proteins and increased proportions of TA were also observed [59]. The PDI values of the WPI-TA complexes also increased in comparison with that of N-WPI (Figure 3B), suggesting a wider particle size distribution of the complexes. However, the PDI values of all N-WPI and WPI–TA samples were less than 0.6, implying a relatively uniform aggregate population formed across the samples [56]. Overall, the particle-size results were consistent with changes in the colloidal organization of the WPI–TA system after TA addition, while the TA-alone controls indicated an important contribution from TA self-association.

### 3.5. Assessment of Binding Between WPI and TA

**Figure 4:**
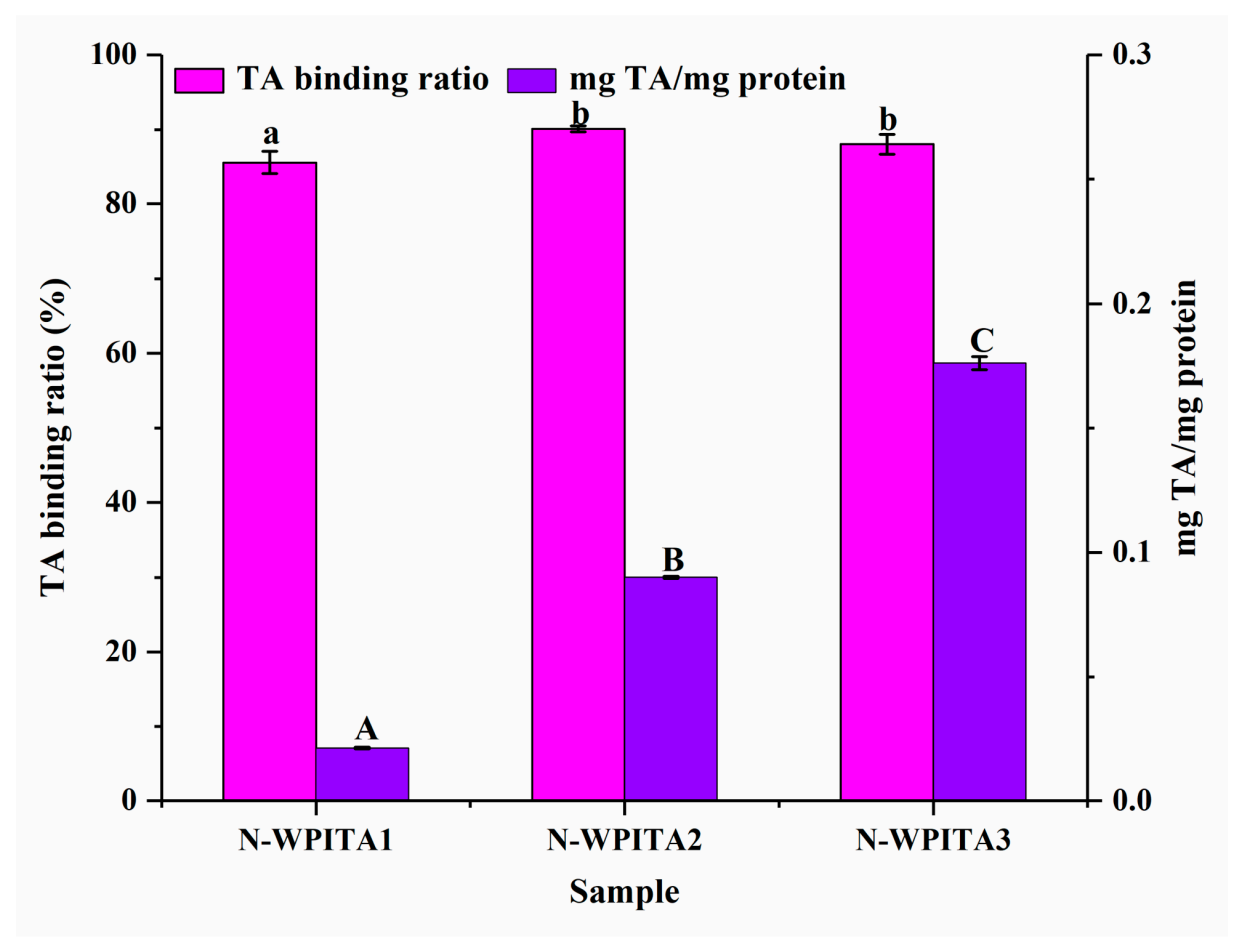
TA binding ratio and TA binding amounts (mg TA/mg protein) of WPI-TA complexes. N-WPI: WPI at 10 mg/mL; N-WPITA1: WPI at 10 mg/mL, TA at 0.25 mg/mL; N-WPITA2: WPI at 10 mg/mL, TA at 1 mg/mL; N-WPITA3: WPI at 10 mg/mL, TA at 2 mg/mL. Differentlowercase letters (a, b) and uppercase letters (A–C) indicate significant differences in the TA binding ratio and the amount of TA bound (mg TA/mg protein) among samples, respectively (*p* < 0.05).

The dialysis behavior of TA and the amount of TA retained in the WPI–TA systems after dialysis were evaluated. The TA removal ratio was evaluated using a TA control at 2.0 mg/mL. After dialysis, the TA concentration remaining inside the dialysis bag decreased from 1.93 ± 0.03 mg/mL before dialysis to 0.02 ± 0.01 mg/mL after dialysis, corresponding to a TA removal ratio of 99.0 ± 0.5% (*n* = 3). These results indicate that freely diffusible TA was effectively removed from the dialysis bag under the conditions used in this study. As shown in Figure 4, the highest estimated TA binding ratio (90.1%) was observed at 1 mg/mL of TA (N-WPITA2) with a slightly lower binding ratio at 2 mg/mL of TA (N-WPITA3). The maximum TA binding amount (0.18 mg/mg protein) was obtained at a TA concentration of 2 mg/mL (N-WPITA3), indicating an association between WPI and TA under the present experimental conditions. Although N-WPITA3 exhibited the largest particle size, this increase cannot be directly attributed to enhanced WPI–TA binding, as TA self-association may also contribute to the observed particle-size changes. As a polyphenol with several active phenolic hydroxyl groups, TA showed an apparent binding capacity toward WPI [22]. However, since the exact galloyl ester composition of the TA preparation was not determined, the binding ratio and binding amount reported here represent the net binding of the heterogeneous TA mixture to WPI. Since the Folin–Ciocalteu assay is not specific to TA, possible interference from WPI cannot be completely excluded. In addition, dialysis may affect the amount of TA retained in the reversible WPI–TA complexes. TA was shown to effectively bind with sodium caseinate [36]. Non-covalent interactions between anthocyanins and soy proteins were also shown to promote their binding with an increasing concentration of anthocyanins, though the maximum binding ratio of anthocyanins occurred at a lower concentration [60]. Although the amount of bound TA continued to increase with increasing TA concentration, the slightly decreased binding ratio at 2 mg/mL suggested that the binding efficiency of WPI toward TA decreased at a higher TA level.

### 3.6. Analysis of Total Free Sulfhydryl Contents

**Figure 5:**
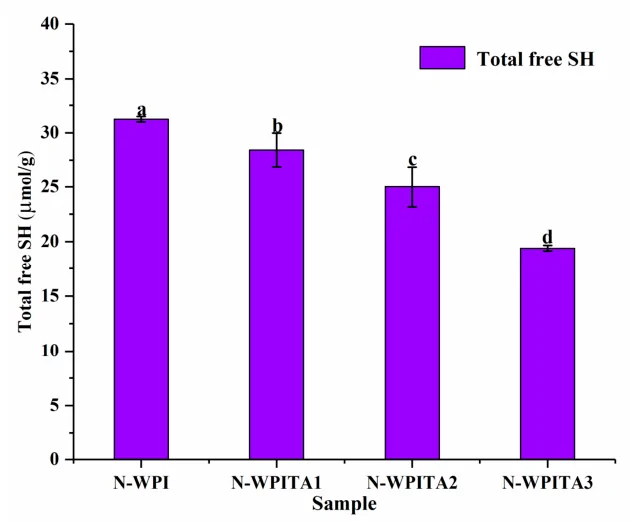
Total free sulfhydryl group content of N-WPI and WPI-TA complexes. N-WPI: WPI at 10 mg/mL; N-WPITA1: WPI at 10 mg/mL, TA at 0.25 mg/mL; N-WPITA2: WPI at 10 mg/mL, TA at 1 mg/mL; N-WPITA3: WPI at 10 mg/mL, TA at 2 mg/mL. Different letters on the error bar indicate significant differences (*p* < 0.05).

The highly chemically active sulfhydryl groups of milk proteins can interact with polyphenolic compounds present in the food matrix, hence influencing the functional properties of the proteins [23,61]. Figure 5 presents the total free sulfhydryl contents of N-WPI and WPI-TA complexes. N-WPI had the highest total free sulfhydryl content (31.2 μmol/g), and significantly decreased contents (*p* < 0.05) were observed for the WPI-TA complexes with an increased proportion of TA, i.e., 28.4 μmol/g (N-WPITA1), 25.0 μmol/g (N-WPITA2), and 19.4 μmol/g (N-WPITA3), suggesting that TA incorporation altered the sulfhydryl environment of WPI. Previously, a significantly decreased content of sulfhydryl groups in the non-covalent complexes formed by whey protein concentrate and quercetin with increased concentration was also reported [50]. Interactions between the sulfhydryl groups of soy protein and the hydroxyl groups of water-soluble anthocyanins were shown to reduce the total free sulfhydryl content in the protein [60]. Similar changes in the sulfhydryl environment of whey proteins after interaction with phenolic derivatives have also been observed [61]. These studies suggest that polyphenol incorporation may affect protein sulfhydryl groups; however, the specific mechanisms may depend on the chemical structures of both proteins and polyphenols [62]. TA alone also showed measurable absorbance under the Ellman assay conditions. After correction for the reagent blank, the A412 values were 0.062 ± 0.002, 0.100 ± 0.002, and 0.180 ± 0.001 for TA at 0.25, 1.00, and 2.00 mg/mL, respectively (*n* = 3). Although this concentration-dependent response suggests that TA itself may make a relatively small contribution to the absorbance measured in the Ellman assay, its potential influence should still be considered. The A412 of TA-alone controls was not subtracted from that of the WPI–TA samples; therefore, the free SH contents of WPI–TA complexes are reported as apparent values. Nevertheless, the concentration-dependent decrease in apparent free SH content suggests changes in the sulfhydryl environment or accessibility of WPI following TA incorporation. Such changes may be associated with altered sulfhydryl accessibility, thiol oxidation, or possible interactions between protein thiols and oxidized phenolic compounds. However, the Ellman assay alone cannot distinguish among these possibilities.

### 3.7. Analysis of Emulsifying Properties

**Figure 6:**
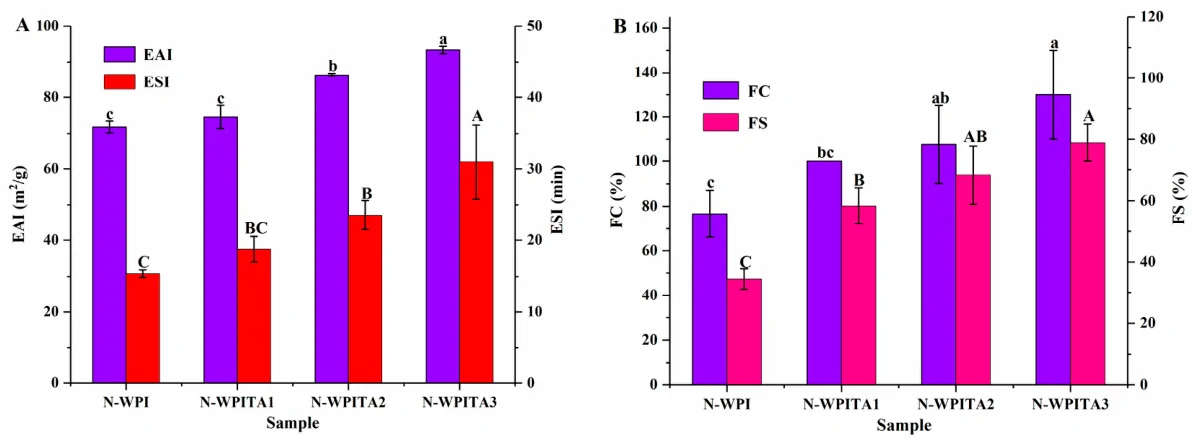
Emulsion activity index and stability index (**A**), and foam capacity and stability (**B**) of N-WPI and WPI-TA complexes. N-WPI: WPI at 10 mg/mL; N-WPITA1: WPI at 10 mg/mL, TA at 0.25 mg/mL; N-WPITA2: WPI at 10 mg/mL, TA at 1 mg/mL; N-WPITA3: WPI at 10 mg/mL, TA at 2 mg/mL. Different letters on the error bar indicate significant differences (*p* < 0.05).

As important emulsifying attributes of proteins, EAI refers to the ability of proteins to adhere at the oil–water interface to decrease the interfacial tension between the oil and water phases, whereas ESI reflects the ability of the formed emulsion to resist gravitational separation and maintain stability [63]. As displayed in Figure 6A, the EAI and ESI of N-WPI were 71.9 m^2^/g and 15.4 min, respectively. Upon non-covalent complexation between WPI and TA, both the EAI and ESI of the WPI-TA complexes increased, to 74.6 m^2^/g and 18.8 min for N-WPITA1, 86.3 m^2^/g and 23.5 min for N-WPITA2, and 93.4 m^2^/g and 31 min for N-WPITA3, respectively. Similar increases in the values of EAI and ESI of WPI were also reported after its non-covalent interactions with polyphenols (gallic acid, chlorogenic acid, and epigallocatechin gallate) [23,64]. These results indicate that the WPI–TA samples exhibited improved emulsifying activity and emulsion stability within the tested TA concentration range. These enhanced emulsifying properties may be associated with the conformational changes and altered interfacial behavior of WPI after interaction with commercial TA constituents. However, the specific contribution of individual molecular interactions requires further investigation. Changes in protein conformation may affect its adsorption behavior at the oil/water interface [35,65]. The interaction between WPI and TA may alter the protein-associated conformational characteristics, which could influence the interfacial behavior and emulsifying properties of WPI [66]. In addition, previous studies have suggested that protein–TA interactions can affect interfacial organization [67]. In the present study, enhanced emulsifying properties were observed within the tested TA concentration range (0.25–2.0 mg/mL). Previous studies have shown that polyphenol concentration can significantly influence protein structure, interfacial properties, and emulsion stability. At excessive polyphenol levels, stronger protein–polyphenol interactions may alter protein aggregation behavior and negatively affect emulsifying properties [68,69]. Whether such effects occur in the WPI–TA system at TA levels above 2.0 mg/mL requires further investigation.

### 3.8. Analysis of Foaming Properties

Dairy products such as cream, yogurt, and ice cream are made up of air bubbles dispersed in the aqueous phase, and it is essential to utilize effective blowing agents that rapidly adsorb at the gas–water interface to prevent the bubbles from merging, disintegration, or expansion [70]. The foaming properties of WPI as affected by interactions with TA were investigated regarding changes in FC, denoting the overall volume of foam generated by a process, and FS, indicating the foam’s capacity to withstand collapse over time [50]. As displayed in Figure 6B, the FC and FS of N-WPI were 76.7% and 34.4%, respectively. In comparison, both the FC and FS of WPI-TA complexes increased by 100% and 58.3% for N-WPITA1, 107.7% and 68.3% for N-WPITA2, and 130% and 78.9% for N-WPITA3, respectively. The enhanced foaming properties may be related to the altered interfacial behavior of WPI after TA addition. However, interfacial adsorption, surface tension, and interfacial film strength were not measured in this study, and the specific mechanism therefore remains to be established. It was reported that the enhanced molecular flexibility of proteins may promote their dispersion at the air–water interface, potentially decreasing surface tension and generating more elastic bubbles at the air–liquid interface [6,50,71]. Similar increases in the values of FC and FS of lactoferrin were also revealed after its non-covalent interactions with procyanidin, which might be ascribed to their higher interfacial elasticity [21]. However, in the work of Zhan et al. [36], the foaming capacity of the TA and sodium caseinate complexes was reduced compared with sodium caseinate, probably due to the decreased surface hydrophobicity of the sodium caseinate molecules. In the present study, enhanced foaming properties were observed within the tested TA concentration range (0.25–2.0 mg/mL). Previous studies have demonstrated that the concentration of polyphenols plays an important role in regulating protein–polyphenol interactions and consequently affects protein functional properties [72]. Therefore, whether further increasing TA concentration would continue to improve or impair the foaming properties of WPI–TA complexes requires further investigation.

## 4. Conclusions

The interaction between WPI and commercial TA was supported by the combined analytical results. The formed WPI–TA complexes exhibited altered protein-associated spectral characteristics after TA incorporation, although the contribution of TA-related absorption should be considered when interpreting spectroscopic results. The decreased total free sulfhydryl content of WPI after TA incorporation suggested changes in the sulfhydryl environment or accessibility of sulfhydryl groups in WPI, although the underlying mechanism requires further investigation. Furthermore, the complexation of WPI with TA increased the mean particle size, reaching a maximum value of 263.4 nm, and resulted in a calculated amount of TA associated with the WPI of 0.18 mg/mg protein at 2 mg/mL TA. The foaming and emulsifying capacities of the WPI-TA complexes were enhanced, whereas the high-temperature thermal transition showed decreased T_onset_ and T_max_ values. Whether higher TA levels may induce aggregation or precipitation and impair these properties, and what the optimal WPI:TA ratio is, remain to be determined. Only three TA concentrations were examined in this study, and both WPI and commercial TA are complex mixtures. Future work could investigate a wider WPI:TA ratio range and examine solubility, zeta potential, and interfacial behavior, with particular emphasis on functional food applications such as foaming, emulsification, encapsulation, and delivery systems. Such studies would help clarify the functional performance of WPI–TA complexes in real food systems.

## Figures and Tables

**Table 1:** Peak positions of selected FTIR bands in N-WPI and WPI–TA samples.

Sample	O–H/N–H Stretching Region	C–H Asymmetric Stretching	Amide I (cm^−1^)	Amide II (cm^−1^)
N-WPI	3293.23 ± 0.47 ^b^	2961.67 ± 1.03 ^a^	1647.02 ± 1.59 ^c^	1535.19 ± 0.44 ^a^
N-WPITA1	3293.80 ± 0.34 ^b^	2962.63 ± 0.88 ^a^	1648.81 ± 0.49 ^bc^	1533.65 ± 1.37 ^a^
N-WPITA2	3294.52 ± 0.64 ^ab^	2961.42 ± 0.65 ^a^	1651.22 ± 0.58 ^ab^	1532.47 ± 1.91 ^a^
N-WPITA3	3295.28 ± 0.67 ^a^	2962.20 ± 0.84 ^a^	1652.99 ± 1.44 ^a^	1532.44 ± 0.22 ^a^
ANOVA *p*-value	0.015	0.310	<0.001	0.07

Values are expressed as mean ± standard deviation (SD) of three independent measurements (*n* = 3). Different superscript letters within the same column indicate significant differences among samples (*p* < 0.05).

**Table 2:** DSC thermal transition parameters of N-WPI and WPI–TA samples, with TA included as a reference.

Sample	T_onset_, 1 (°C)	T_max_, 1 (°C)	T_onset_, 2 (°C)	T_max_, 2 (°C)	T_onset_, 3 (°C)	T_max_, 3 (°C)
N-WPI	46.4 ± 3.8 ^a^	92.6 ± 1.3 ^a^	238.6 ± 0.6 ^a^	244.2 ± 0.4 ^a^	—	—
N-WPITA1	46.6 ± 1.9 ^a^	91.2 ± 0.3 ^a^	232.3 ± 0.4 ^b^	239.79 ± 0.02 ^b^	—	—
N-WPITA2	44.7 ± 3.7 ^a^	91.1 ± 0.5 ^a^	210.4 ± 0.2 ^c^	232.1 ± 0.3 ^c^	—	—
N-WPITA3	47.0 ± 0.5 ^a^	87.4 ± 2.2 ^b^	208.6 ± 0.3 ^d^	225.6 ± 0.4 ^d^	—	—
TA	48.3 ± 2.9	92.6 ± 2.9	189.8 ± 0.6	198.76 ± 0.01	228.7 ± 5.9	238.7 ± 1.0
ANOVA *p*-value	0.756	0.007	<0.001	<0.001	—	—

Values are expressed as mean ± SD (*n* = 3). Different superscript letters within the same column indicate significant differences (*p* < 0.05). Numerical values were rounded according to the precision of the analytical method. TA was included as a reference and was not included in the statistical comparisons with WPI-containing samples.

## Data Availability

The original contributions presented in this study are included in the article. Further inquiries can be directed to the corresponding author.
